# Correction

**Published:** 1854-07

**Authors:** 


The following article was sent us for insertion by a friend, a practicing Dentist, who we presume has himself examined the case to which the article alludes. In a recent letter from Dr. Todd, he informs us that his report of the case—which is reviewed—was given from the description of one of the physicians, either present or who assisted in the operation, and from statements made by friends of the boy. He only saw the boy once, and then his jaw was in bandage so that he could not make a critical examination of the case. But from the assurance that half of the inferior maxillary bone was removed and that new teeth were making their appearance, he naturally inferred that they were productions from new germs supplied by the recuperative powers of the system.—ED.

*Correction.*

*Messrs Editors:* In looking over the Review, of the 17th ult., I was not a little surprised to see, what purports to be the history of a Surgical Operation, with "reproduction of a portion of the lower jaw," etc. Reported for the "Dental Register" of April, 1853, by Dr. J. M. Todd, of Monongahela City, Pa.

This report is so replete with errors of fact, throughout, that I shall have to crave the indulgence of your readers, while I detail a report of the case had in view by Dr. Todd, that can be substantiated.

In the first place, Dr. Keys informs me that he never did, in connection with Dr. Biddle, perform such an operation as the one described by Dr. Todd.

The case, as it occurred, is this :—Wm. Mancha, aged about 12 years, in the autumn of 1850, fell from a straw stack, receiving a slight injury, as was supposed, after which he complained of toothache, for some time, and eventually called on Dr. Shidler, who found the anterior molar of the inferior maxilla, on the left side, so loose that he readily extracted it with dental forceps. This, however, failed to relieve the pain of the jaw; the cheek had also swollen, which continued until an abscess formed, and matter was discharging through the cheek, externally. The case was then variously treated for some months ; the cheek swollen and matter discharged until the spring of 1852, when, at the suggestion of Dr. Keys, who at that time had charge of the case, Dr. Wilson, of Beallsville was consulted, and agreeing with Dr. Keys in opinion, that there was an extensive caries, and that the proper treatment indicated in the premises, was to remove, by an operation, the necrosed bone. In accordance with this opinion, the parents acquiesced, and on the 13th of May the operation was performed by Drs. Keys and Wilson, in presence of the family and two medical students, (Messrs. Bell and Wilson).

The boy was put under the influence of chloroform, a free curvilinear incision was made, from a point anterior to the ear, extending about half way to the mouth, down to the bone, along the region of its base; this brought to view the diseased portion, which was seized with bone forceps, and thereby *wrenched* from its position. After arresting the hemorrhage, the wound being closed was dressed with adhesion strips, and healed kindly. The fracture was oblique, running from a point internally, slightly anterior to the angle, and terminating externally, opposite the tooth extracted, constituting the entire portion (angle, ramus and processes, together with a scale from the outer side of the base) that was removed. Will not Dr. Todd's mountain wonder, that teeth are actually presenting, dwindle into a molehill when it is recollected that *no portion of the jaw containing the rudiments of permanent molars, except perhaps, the dens sapientia, was removed* by the operation ?

In this case, nature has summoned her recuperative energies, and has in a great degree repaired the original loss sustained. The deformity that remains is very perceptible, being a thickening of the cheek from deposition in the cellular tissue during the previous disease, instead of a " little too much bone," and a considerable inclination of the mouth to one side. The motion of the jaw is imperfect. It is, Messrs. editors, through the various medical journals of the day that we are mainly kept posted in our knowledge of the advancement in medical science, and should apochryphal reports be permitted to obtain a place in their columns, then our confidence in their authority will be lost, and we shall be deprived of the benefit of one of our greatest facilities for procuring information. *Verbum sat.* FIAT JUSTITIA

